# 

**Published:** 1900-02-24

**Authors:** 


					THE HOSPITAL. " The standard of highest purity."?Lancet. February 24tii, 1900.
CADBURY'S ff CADBURY'S
COCOA )% ^ M> JgL COCOA
a do/~.t titdt ir nrxDT? NO DRUGS OR ALKALIES USED.
No.^TOy. Vol. XXYII. [S^ Saturday,
Feb. 24, 1900.
[Sutamption Terms,] pK)ICE TWOPENCE.

				

